# The effect of white and green LED-lights on the catch efficiency of the Barents Sea snow crab (*Chionoecetes opilio*) pot fishery

**DOI:** 10.1371/journal.pone.0258272

**Published:** 2021-10-05

**Authors:** Kristine Cerbule, Bent Herrmann, Eduardo Grimaldo, Leif Grimsmo, Jørgen Vollstad

**Affiliations:** 1 Department of Fisheries Technology, SINTEF Ocean, Trondheim, Norway; 2 UiT, The Arctic University of Norway, Tromsø, Norway; 3 DTU Aqua, Denmark Technical University, Hirtshals, Denmark; Maurice Lamontagne Institute, CANADA

## Abstract

In commercial snow crab (*Chionoecetes opilio*) fishery, the catch efficiency of the conical pots is important for increasing the profitability of the industry. This study evaluated the effect of adding green and white light emitting diodes (LED) on the catch efficiency of commercially used conical pots. The results from the field experiments showed that inserting artificial lights significantly increases the catch efficiency for snow crab over the minimum landing size of 95 mm carapace width of up to 76% when using green LED, and by 52–53% on average when using white LED. This study shows that it is possible to improve the catch efficiency of the snow crab fishery by applying artificial LED lights to the conical snow crab pots, potentially resulting in an important economic benefit to the snow crab fishery.

## 1. Introduction

Snow crab (*Chionoecetes opilio*) is a cold-water species that is distributed over the Arctic, inhabiting areas of the north-west Atlantic (Canada and west Greenland) and northern Pacific Oceans [1]. It is an invasive species in the Barents Sea, where its presence was first recorded in 1996 [2] and where a population began to establish between 2004 and 2006 [2]. The commercial fishery for snow crab in the Barents Sea is relatively recent, as it started in 2012, and within three years (2012–2015) the fishery grew very quickly. Norwegian landings increased from 2.5 tonnes in 2012 to 5406 tonnes in 2016 [3], with 11 Norwegian and 18 European Union vessels participating in this fishery [4]. Due to the closure of the Russian part of the Barents Sea to international vessels, in 2017 the landings were reduced to just over 3000 tonnes. In 2018, the snow crab quota in the Norwegian part of the Barents Sea was set to 4000 tonnes. The total catch in 2018 was considerably lower, reaching only 2677 tonnes [5]. However, the catch rates of snow crab started to increase from 2019, and for 2021, the quota is increased to 6500 tonnes [6].

The Norwegian snow crab fishery operates far off the coast at depths of around 200–300 m and uses conical pots. The standard conical pots were introduced to the Barents Sea snow crab fishery from the Canadian east coast [2] because they are convenient during fishing operations, being light, manoeuvrable, and stackable for transportation on fishing vessels to the fishing grounds. Between 2015 and 2019, the catch efficiency rates of the pots in the Barents Sea have varied from 0.5 to 2.5 kg snow crab per pot, with the highest rates in 2015 and 2016 [7]. Since the number of pots used by this fleet is restricted to 9000 units per trip, the focus of the fishery is directed towards increasing the pots’ catch efficiency, reduce the fishing effort (i.e., number of days at sea to fill the quota), associated operational costs and environmental impact [8–10]. Each vessel has the capacity to deploy between 1000 and 2000 pots per day. The Norwegian fishery regulations include a maximum pot soaking time of three weeks, a closed season between the 1^st^ of July and 30^th^ of September, and a maximum of 20% of soft-shell crabs in the total catch [6]. The minimum landing size of the snow crab is 95 mm carapace width (CW) [6] which was reduced from 100 mm CW July 2020 [7]. Therefore, in practice, only mature male crabs can be landed (female snow crabs are rarely larger than 90 mm in CW), and all undersized individuals must be returned to the sea [7]. The pots are baited using squid or herring, and the bait itself is the most expensive operation cost in the fishery, with expenses reaching approximately 20 NOK (equals to approximately 2 EUR) per pot deployment [2]. Therefore, catch efficiency is a major concern for the Barents Sea snow crab fishing industry, and methods for improving it—and thus improving the profitability of the industry—are actively sought.

The use of artificial lights in fisheries has been tested both in terms of increasing catch efficiency by attracting the target species [11–15] and reducing the capture of bycatch species [16–18]. Artificial lights have been applied to different fishing gear. The response behaviour of the target species to the lights is species dependent [19]. Previous research on the effect of light emitting diodes (LED) on the catch efficiency of the snow crab has shown a potential for increased catch efficiency in the snow crab fishery both in the Barents Sea and in Canada [11, 12]. Using white LED lights showed an increase in the catch per unit of effort (CPUE) by up to 77% in experiments carried out in Newfoundland, Canada [11]. However, using white LED lights did not yield significant results in the experiments conducted in the Barents Sea [12]. Thus, more studies are required to determine the effect of using white artificial LED lights on snow crab catch efficiency. In addition, we tested green LED lights. To the best of our knowledge, green LEDs are not tested in earlier trials regarding catch efficiency of the snow crab in the Barents Sea while use of green lights has shown positive results on catch efficiency in snow crab fisheries in Canada [11]. We designed the study to answer the following question: Is there any improvement in catch efficiency if standard conical pots are additionally equipped with artificial white and green LED lights?

## 2. Materials and methods

### 2.1. Ethics statement

All trials carried out during this study followed standard commercial fishing practices and snow crabs were not exposed to any additional harm. Therefore, this study did not require any permits from the authorities. Furthermore, the trials did not involve any endangered or protected species.

### 2.2. Sea trials and data collection

Sea trials were carried out on board the commercial fishing vessel “Northeastern” (overall length 55.2 m and 2250 HP) which operates 9000 pots and has a capacity of deploying and retrieving an average of 2000 pots per day. Comparative fishing experiments were conducted from 1^st^ to 24^th^ of May 2020 in the Barents Sea (latitude between N75°4262 and N76°1160; longitude between E33°1789 and E36°7520) (ICES area 1.b). The depth of the fishing grounds varied between 250 and 280 m.

### 2.3. Pots and experimental design

During the sea trials, the same conical snow crab pots used in the commercial fishery operating in the Barents Sea and along the east coast of Canada were used [9, 20]. The dimensions of the pots were 130 cm in diameter at the bottom and 55 cm at the top, where the entrance to the pot is located. The height of the pots was 60 cm. The pot frame and the lower ring were made of 12-mm and 14-mm steel bars, respectively, yielding a pot weight of approximately 12.5 kg. Diamond mesh netting (mesh size 140 mm) made of Ø4 mm single braided polyethylene (PE) twine was used (mesh opening angle ranges from 64° to 89° [10]). The 140 mm netting has a CW50 of approx. 95 mm [8], meaning that 50% of legal-size snow crabs have the probability of escaping or being retained. The pots were attached to longlines by a quick link system with at least a 30 m distance between each pot.

Pots with and without lights, herein called test (T) and baseline (B) pots, respectively, were deployed on the same longline. After every second or third B pot there was a T pot.

Three series of experiments were carried out during the sea trials using lights. Pots in each of the series were linked on one separate longline resulting in three deployments of longlines:

1. Adding white lights to standard pots with a pot soaking time of 8 days. Each T pot with a LED light was followed by three B pots (T-B-B-B-T-B-B-B-T…);
2. Adding white lights to standard pots with a soaking time of 14 days. Each T pot with a LED light was followed by two B pots (T-B-B-T-B-B-T…);
3. Adding green lights to standard pots, extending the soaking time to 17 days. Each T pot with a LED light was followed by two B pots (T-B-B-T-B-B-T…).

Therefore, each of the series consisted of pots connected to one separate longline each. The pots of the three series were not fished simultaneously; however, they were deployed during the same fishing trials using the same snow crab fishing vessel. The experiments were conducted on board commercial snow crab fishing boat; therefore, the fishing area of the line deployments were slightly different and dependent on conditions of the vessel operation, weather conditions and drift ice. Therefore, it was not possible to keep similar soaking time conditions for all three experiments.

**Fig 1:**
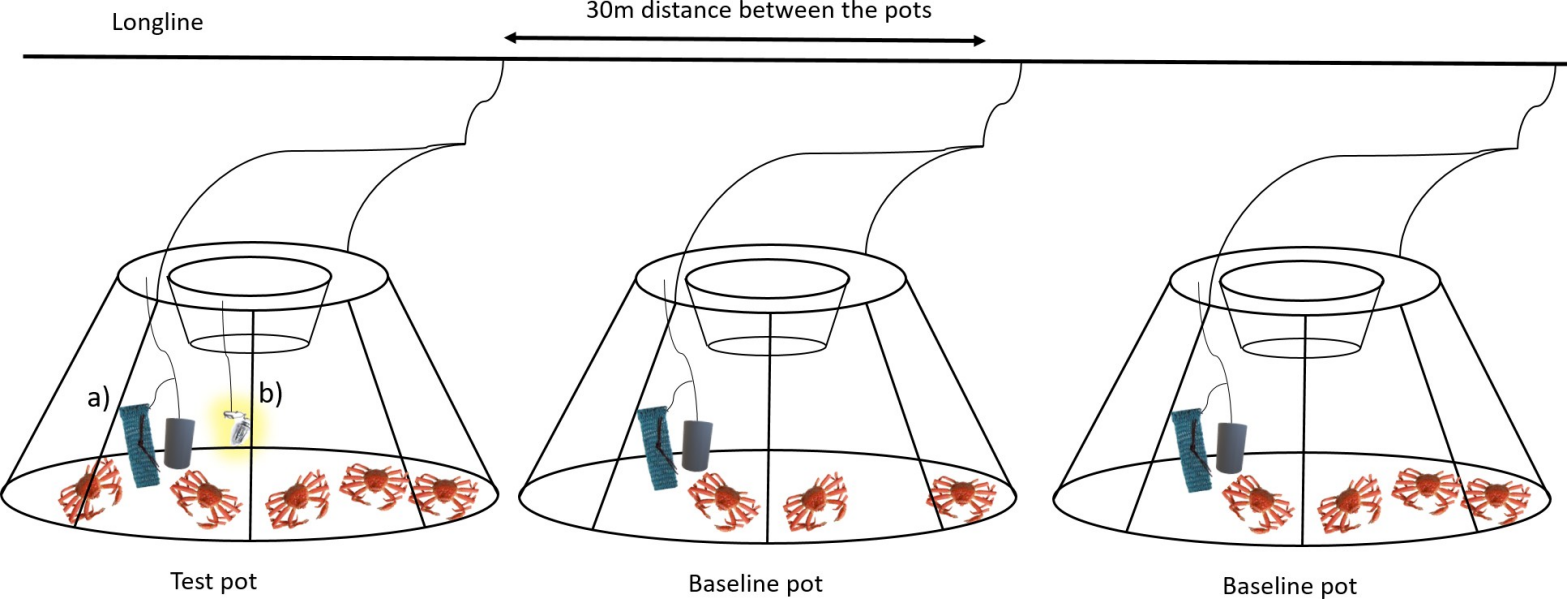
Distances between standard conical pots with top entrance, location of the bait (a) and artificial LED light used for the test pots (b).

Each pot was baited with approximately 2 x 400 g of squid (*Illex spp*.). Half of the bait was placed in a bait container and the other half in a bait bag, which was mounted under the entrance of the pot (Fig 1). White and green PotLight LED fishing lights—manufactured by FishTekMarine [21]—were used in this experiment. The lights have a maximum operation depth of 1200 m and a battery life of approximately 500 hours.

When the pots were hauled on board, they were emptied separately onto a sorting board where the CW of each snow crab was measured to the nearest millimetre using a calliper according to Jadamec et al. [22]. The number of T and B pots that did not retain any snow crab was also recorded.

### 2.4. Estimation of the effect of light on the snow crab catch efficiency

The estimation of the absolute catch efficiency for both B and T pots requires data on the abundance and size of the snow crab population present in the fishing grounds at the time and position where the pots are being deployed, and these data were not available in this study. Moreover, the relationship between the absolute catch efficiency of each pot and pot soaking time is unknown [9]. Furthermore, modelling the total CPUE for pots with and without lights would provide only a limited general estimate, as CPUE depends on the spatial and temporal availability of snow crab, which varies with time and fishing ground [9].

In contrast with the absolute catch efficiency, the relative catch efficiency between two pot setups can be estimated based on catch data from the fishing experiments. This approach does not require information on the size-dependent availability of snow crab in the fishing ground. To estimate the relative catch efficiency between the two pot setups, both should be deployed simultaneously in the same fishing ground with the same soaking time and bait amount. Therefore, we used the relative size-dependent catch efficiency between the two pot designs to isolate and quantify the effect of adding LED-lights to some of the pots. Estimation of the relative catch efficiency is a well-established method that has been widely used for comparing catch efficiency of passive fishing gear [23–27]. We used the statistical software SELNET for the analysis of the data [28].

### 2.5. Modelling the relative size-dependent catch efficiency between pots with and without LED-lights

The relative size-dependent catch efficiency between pots with and without LED-lights was independently estimated for each of the three experimental trials.

To assess the relative size-dependent catch efficiency derived from adding LED lights to the pots, we used unpaired catch comparison and catch ratio analyses [23]. We were interested in the size-dependent catch comparison rate values summed over the deployments of both T and B pots (deployment is equivalent to the number of baseline or treatment pots). During the experimental fishing trials, the catch data obtained for both types of pots were not collected in pairs, nor did the data correspond to the same total number of deployments, since each T pot was followed by two or three B pots on each longline. The catch data from the deployments of both pot types were summed and compared with the total deployment data by minimising Eq (1) to estimate the functional form of the summed catch comparison rate (the experimental rate being expressed by Eq (2) for T versus B pots). Eq (1) is given by:

$$-\sum_{w}\{\sum_{i=1}^{tq}\left[{nt}_{wi}\times ln(CC(w,v))\right]+\sum_{j=1}^{bq}\left[{nb}_{wj}\times ln(1.0-CC(w,v))\right]\}$$ (1)

where ***v*** is a vector representing the parameters of the function describing the catch comparison curve defined by *CC*(*w*,***v***), and *nt*_*wi*_ and *nb*_*wj*_ are the numbers of snow crabs measured in each CW class *w* for T and B pots, respectively. In Eq (1), *tq* and *bq* are the number of deployments carried out with T and B pots, respectively, and the inner summations in the equation represent the sum of the total deployment data. The outer summation in the equation is the summation over the size classes *w*. Eq (1) is equivalent to maximising the likelihood for the observed data based on a maximum likelihood formulation for binominal data [29]. This maximization problem is turned to minimisation problem by putting a minus sign before the Eq (1). The experimental summed catch comparison rate, *CC*_*w*_, where *w* denotes the snow crab CW, is given by Eq (2):

$${CC}_{w}=\frac{\sum_{i=1}^{tq}{nt}_{wi}}{\sum_{i=1}^{tq}{nt}_{wi}+\sum_{j=1}^{bq}{nb}_{wj}}$$ (2)

When both the catch efficiency of T and B pots and the number of deployments are equal (i.e., *tq* = *bq*), the expected value for the summed catch comparison rate would be equal to 0.5. In this case the presence of unequal numbers for T and B pots in each series, *tq*/(*tq* + *bq*) would be used to judge whether or not there is a difference in catch efficiency between the pots. The experimental *CC*_*w*_ is often modelled by the function *CC*(*w*,***v***), which has the following form [30]:

$$CC(w,v)=\frac{exp(f(w,v_{0},\ldots ,v_{k}))}{1+exp(f(w,v_{0},\ldots ,v_{k}))}$$ (3)

In Eq 3, *f* is a polynomial of order *k* with coefficients from *v*_*0*_ to *v*_*k*_. We considered an *f* of up to an order of 4 with parameters *v*_*0*_–*v*_*4*_. Leaving out one or more of the parameters *v*_*0*_…*v*_*4*_ led to 31 additional models that were also considered as potential models for the catch comparison *CC*(*w*,***v***) between T and B pots. Among these models, estimations of the catch comparison rate were made using a multi-model inference to obtain a combined model [23, 31].

The ability of the combined model to describe the experimental data was evaluated based on the *p*-value, which quantifies the probability of obtaining by coincidence at least as big a discrepancy between the experimental data and the model as the one observed. Therefore, this *p*-value, which was calculated based on the model deviance and the degrees of freedom, should not be <0.05 for the combined model to describe the experimental data sufficiently well [32].

Based on the estimated catch comparison function *CC*(*w***, *v***) we obtained the relative catch efficiency (also named catch ratio) *CR*(*w*, ***v***) between the pots with and without LED lights, and described by the following relationship [23]:

$$CR(w,v)=\frac{qb\times CC(w,v)}{qa\times (1-CC(w,v))}$$ (4)

The catch ratio is a value that represents the ratio between the catch efficiency of pots with and without LED lights. Thus, if the catch efficiency of both pot types is equal, *CR*(*w*,***v***) should always be 1.0. Thus, *CR*(*w*,***v***) = 1.5 would mean that the T pots with added LED lights are catching 50% more snow crab with size *w* than the B pots. In contrast, if *CR*(*w*,***v***) = 0.5, it would mean that the T pots are only catching 50% of snow crabs with size *w* that the B pots are retaining.

The confidence limits for the catch comparison curve and catch ratio curve were estimated using a double bootstrapping method [23]. The procedure accounted for the variation between pots in terms of snow crab availability and catch efficiency by selecting *tq* pots with replacement from the pool of T pots, and *bq* pots with replacement from the pool of B pots deployed during each bootstrap repetition. Uncertainty in the size structure of the catch data within the pots was accounted for by randomly selecting crabs with replacement from each of the selected pots separately. We performed 1000 bootstrap repetitions and calculated the Efron 95% [33] confidence limits for the catch comparison and catch ratio curve.

To identify the snow crab sizes associated with a significant difference in pot catch efficiency, we checked for size classes in which the confidence limits for the combined catch comparison curve did not contain *tq*/(*tq* + *bq*) and in which the confidence limits for the catch ratio curve did not contain the value 1.0.

### 2.6. Estimation of size-integrated catch ratio

Size-integrated average values (in percentage) for the catch ratio (*CR*_*average*_) were estimated directly from the experimental catch data by the following equations:

$$\begin{array}{c} {CR}_{average-}=100\times \frac{qb\times \sum_{w<mw}\sum_{i=1}^{tq}{nt}_{wi}}{qa\times \sum_{w<mw}\sum_{j=1}^{bq}{nb}_{wj}}\\ {CR}_{average+}=100\times \frac{qb\times \sum_{w\geq mw}\sum_{i=1}^{tq}{nt}_{wi}}{qa\times \sum_{w\geq mw}\sum_{j=1}^{bq}{nb}_{wj}} \end{array}$$ (5)

where the outer summations include the size classes measured in the catch during the experimental fishing period under (for *CR*_*average*−_) and over (for *CR*_*average*+_) the minimum landing size (*mw* = 95 mm CW), respectively.

To investigate how well the size selectivity of the T and B pots matches with the size structure of the snow crab population present in the fishing ground, two fishing sustainability indicators (*DiscardRatio*_*Test*_ and *DiscardRatio*_*Baseline*_) were estimated directly from the experimental catch data using the following equations:

$$\begin{array}{c} {DiscardRatio}_{Test}=100\times \frac{\sum_{w<mw}\sum_{i=1}^{tq}{nt}_{wi}}{\sum_{w}\sum_{i=1}^{tq}{nt}_{wi}}\\ {DiscardRatio}_{Baseline}=100\times \frac{\sum_{w<mw}\sum_{j=1}^{bq}{nb}_{wj}}{\sum_{w}\sum_{j=1}^{bq}{nb}_{wj}} \end{array}$$ (6)

where the outer summations include the size classes present in the catch during the experimental fishing period under (in nominator) and over (in denominator) all snow crab sizes. The *DiscardRatio* indicators quantify the ratio between undersized and target-size captured snow crabs (snow crab below and above *mw*, respectively). Ideally their value should be a as low as possible. *DiscardRatio* values are both affected by the size selectivity of the pots and by the size structure of the crab population present in the fishing grounds, and therefore provide an estimate that is specific for the targeted population and that cannot be extrapolated to other areas and seasons.

We used this equation to provide a size-averaged value for the effect of adding green or white LED lights on the catch efficiency of the pots. In contrast to the size-dependent evaluation of the catch ratio, *CR*_*average*_ is specific for the snow crab population structure encountered during the experimental sea trials, and it cannot be applied to other scenarios in which the size structure of the population may be different, depending on time period and fishing area [9].

### 2.7. Catch pattern analysis

Finally, size frequency distribution and cumulative size frequency distribution analyses were used. The method provides the proportion of the total catch up to a given size for the T and B pots. The analysis was carried out for T and B pots using the following equation:

$$C{Dn}_{W}=\frac{\sum_{i=1}^{q}\sum_{w=0}^{W}n_{iw}}{\sum_{i=1}^{q}\sum_{w}n_{iw}}$$ (7)

where *n*_*wi*_ is the number of snow crabs of size class *w* in pot *i* (T or B pot, respectively). Based on this information, the cumulative size frequency distribution *CDn*_*W*_ was obtained by the summations of *i* and *w* in Eq (7) over the *q* number of pots deployed during the experimental trials and CW size class *w*, respectively. *CDn*_*W*_ quantifies the proportion (in number of snow crabs) of a total catch reaching a given size class *W*. By incorporating the evaluation of Eq (7) in the double bootstrap described above we obtained the confidence intervals for *CDn*_*W*_.

## 3. Results

We deployed 174 pots in series 1 (white LED with 8 days soak time) and 2 (white LED with 14 days soak time), and 135 pots in series 3 (green LED with 17 days soak time) (Table 1). Each of the experimental series contained 48 to 54 T pots and 81 to 126 B pots, and for these, the CW of all captured snow crabs was measured (Table 1). The number of snow crabs in T and B pots varied between the three experiments (series 1–3). In total, 1583 crabs were measured for the T pots and 2065 for the B pots; therefore, a total of 3648 measurements were included in this study.

**Table 1:** Experimental data sets of the three experiments.

Series	Soaking time (days)	Latitude	Longitude	Depth (m)	Number of baseline pots	Snow crabs in baseline pots	Number of test pots	Snow crabs in test pots
**White light**	8	N75°4262	E33°1789	260	126 (9)	413 (96)	48 (0)	270 (86)
**White light**	14	N76°1160	E36°7520	280	120 (2)	891 (122)	54 (2)	432 (43)
**Green light**	17	N75°4430	E33°2089	250	81 (3)	761 (73)	54 (0)	881 (88)

Corresponding start position of the lines, depth, soaking time, number of pots used for carapace width measurements in test and baseline pots, and number of snow crabs retained in test and baseline pots in each experiment. Number in parentheses for the snow crab shows the amount of snow crab under minimum landing size of 95 mm CW. Number in parentheses for the pots shows the number of empty pots.

The fit statistics obtained showed that the deviation between the experimental catch comparison rate and the modelled curve could be a coincidence, as the *p*-value for all three experiments was larger than 0.05 (Table 2). Therefore, the model can be used to assess the effect on catch efficiency derived from adding artificial lights to the standard commercial pots used as the baseline.

**Table 2:** Catch ratio (*CRw*) and fit statistics obtained for test pots using artificial lights against baseline pots without lights according to Eq (6).

	White light, 8 days soaking time	White light, 14 days soaking time	Green light, 17 days soaking time
***p*-value**	0.1547	0.1246	0.0895
**Deviance**	76.55	84.89	79.67
**DOF**	65	71	64
***CRaverage-***	172.92 (120.36–238.01)	67.38 (38.73–108.30)	189.09 (122.00–295.90)
***CRaverage+***	152.96 (120.26–198.50)	152.41(121.09–191.86)	176.38 (145.72–216.07)
***DiscardRatio*_*test*_**	31.85 (25.00–39.36)	9.98 (6.37–14.36)	10.17 (7.73–13.04)
***DiscardRatio*_*baseline*_**	29.25 (23.05–36.27)	20.04 (14.77–25.79)	9.55 (6.74–12.68)

Values in parentheses represent the 95% confidence limits. DOF = degrees of freedom.

**Fig 2:**
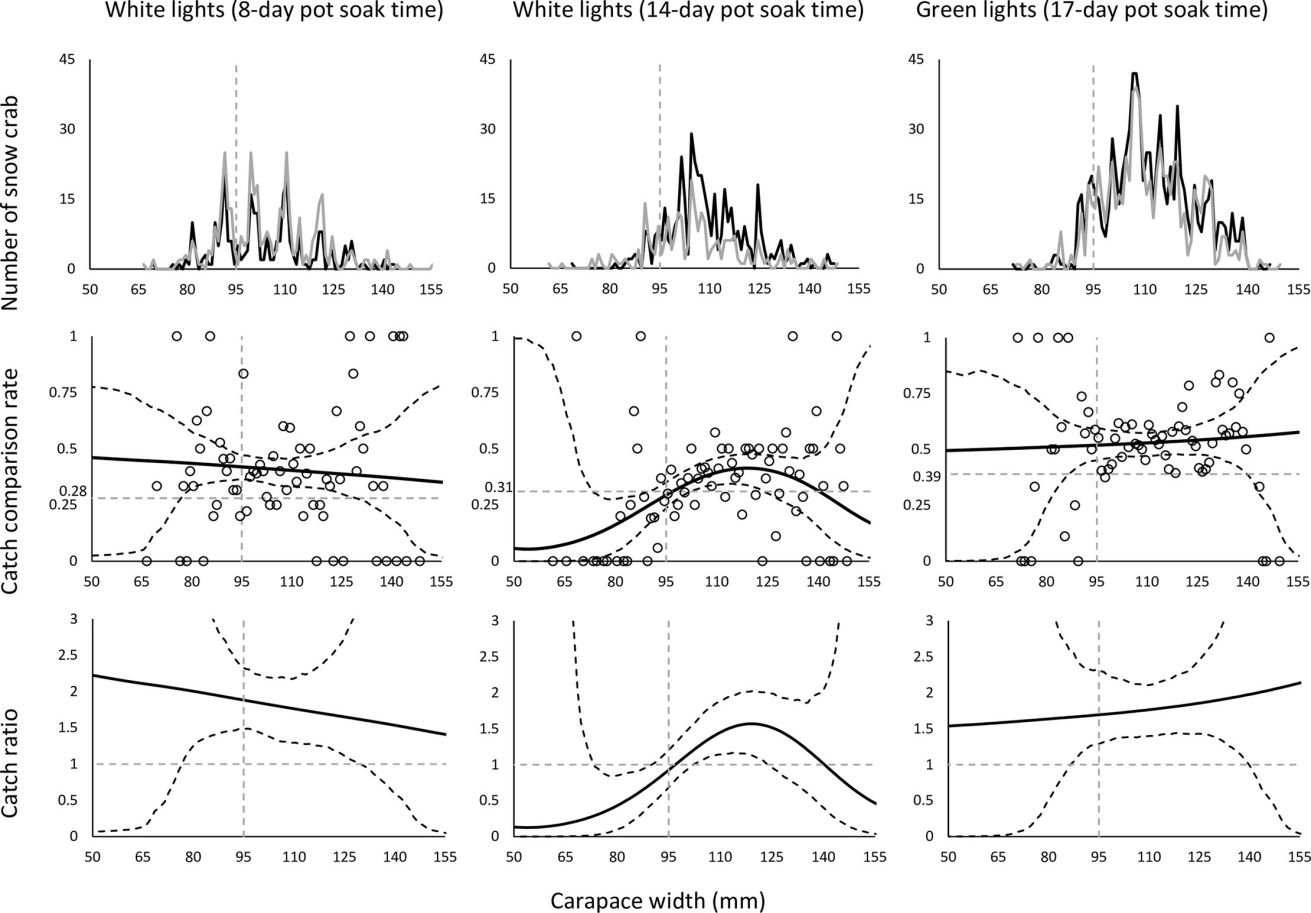
**Population caught in test (black line) and baseline (grey line) pots (upper row), catch comparison rates (middle row), and catch ratios (bottom row) for baseline pots against test pots using white LED lights (left column– 8-day soaking time; middle column– 14-day pot soaking time) and green LED (left column).** Circle marks represent experimental catch comparison rates. The black solid curve in catch comparison and catch ratio plots represents the mean estimated curve and the stippled lines represent the 95% confidence intervals. Horizontal stippled grey lines represent the baseline for the absence of any significant effect of adding artificial lights on catch comparison rate and catch ratio. The grey stippled vertical line represents the minimum landing size for snow crabs (95 mm CW).

In the first series where white LED lights were used for pots with an 8-day soaking time, the catch efficiency increased significantly (i.e., 52.96% for crabs above the minimum landing size (*CR*_*average+*_ = 152.96 (CI: 120.26–198.50)) (Table 2; Fig 2). Both T and B pots retained 32% (*DiscardRatio*_*test*_ = 31.85 (CI:25.00–39.36)) and 29% (*DiscardRatio*_*test*_ = 29.25 (CI:23.05–36.27)) of undersized crabs, respectively.

In the second series with white light and 14-day soaking time, the *DiscardRatio* was significantly lower when the pot soaking time was increased from 8 to 14 days (Table 2). The percentage of individuals under minimum landing size was 10% (*DiscardRatio*_*test*_ = 9.98 (CI:6.37–14.36)) on average for the T pots with white lights and 20% for the B pots (*DiscardRatio*_*baseline*_ = 20.04 (CI:14.77–25.79)). In addition, the T pots showed a significant increase in the retention of crabs with CW over 95 mm (*CR*_*average+ =*_ 152.41 (CI:121.09–191.86)), while indicating less capture of crabs under this size (*CR*_*average-*_ = 67.38 (CI:38.73–108.30)) compared to the B pots. between T and B pots (Table 3).

**Table 3:** Catch ratio (*CR* (*w*)) (%) according to Eq (4).

*w*(mm)	*CR* (*w*) (%) to baseline pot		
	White light (8 days)	White light (14 days)	Green light (17 days)
**70**	207.42 (43.05–532.68)	24.42 (1.18–139.49)	160.12 (6.19–583.05)
**75**	203.88 (89.77–435.80)	32.92 (3.67–88.67)	161.92 (19.50–445.81)
**80**	200.05 (127.76–344.98)	44.25 (10.76–85.38)	163.74 (54.69–332.58)
**85**	196.03 (139.49–303.25)	58.56 (25.42–90.28)	165.59 (92.84–265.86)
**90**	191.91 (145.39–258.85)	75.57 (46.94–101.34)	167.51 (119.48–234.99)
**95**	187.80 (148.81–231.34)	94.44 (71.20–122.71)	169.54 (129.69–230.09)
**100**	183.74 (142.18–221.52)	113.81 (93.70–148.40)	171.71 (137.04–219.71)
**105**	179.78 (131.58–219.32)	131.76 (108.04–173.60)	174.05 (138.11–212.09)
**110**	175.90 (129.60–219.00)	146.13 (114.34–189.07)	176.61 (139.96–211.16)
**115**	172.12 (127.12–228.53)	154.88 (115.61–199.46)	179.41 (142.95–218.67)
**120**	168.40 (122.70–246.79)	156.56 (109.97–201.58)	182.48 (142.94–227.94)
**125**	164.70 (109.60–279.55)	150.68 (94.46–198.55)	185.87 (142.92–248.42)
**130**	160.98 (98.27–322.10)	137.92 (77.41–190.78)	189.59 (136.35–280.38)
**135**	157.16 (79.78–352.66)	119.94 (59.82–186.37)	193.68 (120.98–338.71)
**140**	153.17 (57.834–418.90)	99.13 (38.07–208.94)	198.17 (95.19–520.15)
**145**	148.98 (28.09–584.23)	78.17 (19.43–335.40)	203.08 (54.91–969.14)
**150**	144.70 (9.33–779.89)	59.61 (8.52–975.47)	208.42 (17.54–1834.37)

Values in parentheses represent 95% confidence intervals.

In the third series using green LED lights and 17-day soaking time, the catch efficiency of the pots for snow crabs with CW above the minimum landing size increased significantly by 76% (*CR*_*average+*_ = 176.38 (CI:145.72–216.07)) (Table 2; Fig 2). The retention of undersized crabs did not differ significantly when using green LED lights, and was approximately 10% and 20% in T and B pots, respectively (Table 3).

**Fig 3:**
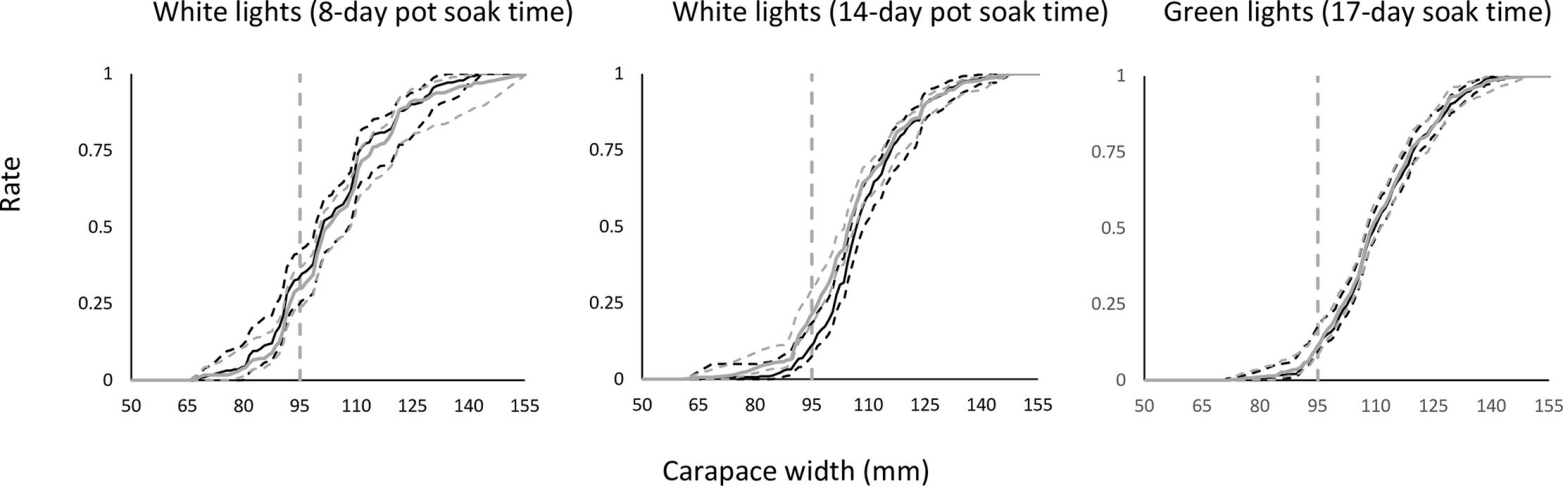
**Cumulative size frequency distribution curves for baseline pots (grey line) and light experiments (black line) using (left to right): White lights (pot soak time 8 days), white lights (soak time 14 days), and green lights (soak time 17 days).** The stippled curves are the 95% confidence limits. The grey stippled vertical line represents the minimum landing size (95 mm CW).

The cumulative size frequency distribution plots show that the catch of both T and B pots consisted of a larger proportion of snow crabs above the minimum landing size in all three series (Fig 3). The comparison of catch patterns *CDn*_*W*_ between the pots with and without lights did not show clear significant differences in any of the three series (Fig 3). For pots with green lights, only 10–12% of the snow crabs were undersized. For pots with white lights, the proportion of undersized snow crabs was around 30%, but it was reduced to 12–20% as the soaking time of these pots was increased from 8 to 14 days. (Fig 3).

## 4. Discussion

In this study, we investigated the effect of adding white and green LED lights on the catch efficiency of conical snow crab pots in the Barents Sea snow crab fishery. Our results showed significant differences in catch efficiency between pots with and without LED lights. The increase in catch efficiency when green lights were used was 76% on average for crabs over minimum landing size. The results showed a significant increase in catch efficiency also when using white LED lights, specifically for the capture of snow crabs above the minimum landing size, which increased by 53 and 52% on average, for pots with soaking time of 8 and 14 days, respectively.

Artificial lights have been tested in different fishing gear to increase the catch efficiency and selectivity of the fishery [11–18]. However, the results are species-specific [19]. The underlying mechanism to allow explaining these results regarding the behavioural response of snow crab towards artificial light in this and earlier studies is not clear [19]. Earlier trials have shown varying results regarding the catch efficiency of snow crab pots when using artificial lights in Newfoundland, Canada and in the Barents Sea [11, 12]. Experiments in the Barents Sea [12] did not detect significant increase in catch rate of snow crab when using white LED as contrary to experiments conducted in Canada [11] and in this study. Different factors might have affected the results of the observed effect on the catch efficiency, such as different area where the trials are conducted, differences in seasons and the type of the lights used.

Our results were consistent with the findings of Nguyen et al. [11, 12], that reported an increase in catch efficiency when snow crab pots were equipped with different type of LED lights. However, the use of the particular light colours—white and green LED lights—has previously been reported as not having a significant effect on catch efficiency in snow crab fishery in the Barents Sea [12] and in laboratory experiments in Canada [11], respectively. It is unclear why our results show a significant increase in capture efficiency compared to those from Nguyen et al. [11, 12]. A possible explanation could be the difference in the intensity of the lights, as Nguyen et al. [11, 12] used Electralume® fishing lights produced by Lindgren Pitman while PotLight [21] fishing lights were used in this study. Moreover, Nguyen et al. [11] used a CPUE method which is strongly affected by snow crab abundance which leads to higher uncertainties in estimation as explained in the beginning of the section regarding estimation of the effect of light on the snow crab catch efficiency. The catch comparison analysis used in this study, is not affected by the abundance to the same extent.

Additionally, our results showed a negative correlation between the retention of undersized snow crabs (*DiscardRatio*) and the soak time, with T pots of series 1 retaining 29–32% of snow crabs under the minimum landing size. The same result was not shown in series 2 and series 3 (Table 2). This can be explained by longer pot soak time in series 2 (14 days) when compared to the series 1 (8 days). Increasing the pot soaking time leads to improved size selectivity, as more undersized snow crabs are able to escape the pot [10, 34]. However, we did not make a direct comparison of the effect of soak time between the pots of different series in this study. Additionally, we are not sure about the cause regarding why baseline pots soaked 14 days contained a higher number of undersized snow crab. The correlation estimated in our study can be affected by the snow crab distribution in the specific position where the pots are deployed, since the *DiscardRatio* estimation is specific for the snow crab population structure encountered.

The experiments with the pots were carried out by conducting one fleet deployment containing more than 100 pots each for each treatment in a specific period of the year (May). This aspect could raise concerns regarding how representative these results are for the snow crab fishery in general. However, the time of the year and the area in which the experiments were carried out represent typical conditions for the commercial snow crab fishery.

This study demonstrated that the addition of LED lights can significantly increase the catch efficiency of the snow crab pots used in commercial fishing. The distance between pots in the present study was kept at approximately 30 m following the practice in commercial fishery. However, the light intensity that can attract the crabs to the pots is unknown. This may question independency between the test and baseline pots of the experimental design by a possible light contamination from the test to the baseline pots. If LED light in the test pots affect the baseline pots by increasing the probability of capturing crabs close to the pots, then the effect on the baseline pots close to the test pots would be negative as a crab would have a higher probability to move towards the more illuminated test pots. This requires that the light intensity for the LED source is high enough not to be almost depleted by 30 m travel in the seawater. On the other hand, if the depletion distance is much higher than 30 m, the use of LED lights could also positively affect the catches in the baseline pots. However, the effect in the test pots will still be higher as light intensity is highest. Unfortunately, for the LED lights that were used in these trials, the depletion distance in seawater was not measured. Therefore, future studies should consider using longer distances between individual pots on the line.

Increase in catch efficiency for pots equipped with green LED lights as obtained in this experiment represents a substantial profitability incentive to the snow crab fishery. No significant difference in catch efficiency between green and white LED lights were shown since the confidence bands overlap between the different treatments. However, more systematic tests using more replicates of longlines with pots, using different light types within different seasons and snow crab fishing areas are needed to apply those results. Additional experiments using video recordings would help for increasing the understanding of snow crab behaviour in presence of artificial lights. Likewise, a comprehensive economic analysis should be carried out in order to evaluate the cost-benefit effects of fishing with light-equipped pots.

## Supporting information

S1 File S1-S6 Snow crab measurements. Data for individual pots. The data consists of snow crab carapace width (mm) measurements for test and baseline pots in each of the three series.
(ZIP)
